# Supplementary material for: Correlation of Bronchoscopy and CT in Characterizing Malignant Central Airway Obstruction
Source: Cancers (Basel). 2024 Mar 23;16(7):1258. doi: 10.3390/cancers16071258 (PMC11010868; doi:10.3390/cancers16071258)
Supplement: Supplementary file 1 [file cancers-16-01258-s001.zip › cancers-2928351-supplementary.pdf]

Supplemental Table S1. Subtype of Obstruction in CT vs. Bronchoscopy in the Bronchoscopy Identified Cohort.

		Bronchoscopy			
		Intrinsic	Mixed	Extrinsic	Total (CT)
**CT**	Intrinsic	**7**	0	0	7
	Mixed	2	**28**	7	37
	Extrinsic	0	1	**9**	10
	Total (Bronchoscopy)	9	29	16	**54**

Details of subtype of obstruction as identified by CT vs. Bronchoscopy in the bronchoscopy identified cohort. Percentage of agreement between the two modalities was 81% (44/54). Cohen's κ = 0.667, $p < 0.001$. CT = Computed Tomography.

Supplemental Table S2. Subtype of Obstruction in CT vs. Bronchoscopy in the CT Identified Cohort.

		Bronchoscopy			
		Intrinsic	Mixed	Extrinsic	Total (CT)
**CT**	Intrinsic	**8**	1	1	10
	Mixed	0	**17**	6	23
	Extrinsic	0	2	**15**	17
	Total (Bronchoscopy)	8	20	22	**50**

Details of subtype of obstruction as identified by CT vs. Bronchoscopy in the CT identified cohort. Percentage of agreement between the two modalities was 80% (40/50). Cohen's $\kappa = 0.685$, $p < 0.001$. CT = Computed Tomography.

Supplemental Table S3. Degree of Obstruction in CT vs. Bronchoscopy in the Bronchoscopy Identified Cohort.

		Bronchoscopy			
		25 - 50%	51 - 75%	76 - 100%	Total (CT)
CT	25 - 50%	**4**	6	0	10
	51 - 75%	4	**0**	4	8
	76 - 100%	1	2	**33**	36
	Total (Bronchoscopy)	9	8	37	**54**

Details of degree of obstruction as identified by CT vs. Bronchoscopy in the bronchoscopy identified cohort. Percentage of agreement between the two modalities was 69% (37/54). Quadratic κ = 0.692, p <0.001. CT = Computed Tomography.

Supplemental Table S4. Degree of Obstruction in CT vs. Bronchoscopy in the CT Identified Cohort.

		Bronchoscopy			
		25 - 50%	51 - 75%	76 - 100%	Total (CT)
**CT**	25 - 50%	**6**	5	2	13
	51 - 75%	3	**5**	3	11
	76 - 100%	0	6	**19**	25
	Total (Bronchoscopy)	9	16	24	**49**

Details of degree of obstruction as identified by CT vs. Bronchoscopy in the bronchoscopy identified cohort. Percentage of agreement between the two modalities was 61% (30/49). Quadratic κ = 0.607, p <0.001. CT = Computed Tomography.
